# Appropriateness of Overridden Alerts in Computerized Physician Order Entry: Systematic Review

**DOI:** 10.2196/15653

**Published:** 2020-07-20

**Authors:** Tahmina Nasrin Poly, Md.Mohaimenul Islam, Hsuan-Chia Yang, Yu-Chuan (Jack) Li

**Affiliations:** 1 Graduate Institute of Biomedical Informatics College of Medical Science and Technology Taipei Medical University Taipei Taiwan; 2 International Center for Health Information Technology (ICHIT) Taipei Medical University Taipei Taiwan; 3 Research Center of Big Data and Meta-Analysis Wan Fang Hospital Taipei Medical University Taipei Taiwan; 4 Department of Dermatology Wan Fang Hospital Taipei Medical University Taipei Taiwan; 5 TMU Research Center of Cancer Translational Medicine Taipei Medical University Taipei Taiwan

**Keywords:** clinical decision system, computerized physician order entry, alert fatigue, override, patient safety

## Abstract

**Background:**

The clinical decision support system (CDSS) has become an indispensable tool for reducing medication errors and adverse drug events. However, numerous studies have reported that CDSS alerts are often overridden. The increase in override rates has raised questions about the appropriateness of CDSS application along with concerns about patient safety and quality of care.

**Objective:**

The aim of this study was to conduct a systematic review to examine the override rate, the reasons for the alert override at the time of prescribing, and evaluate the appropriateness of overrides.

**Methods:**

We searched electronic databases, including Google Scholar, PubMed, Embase, Scopus, and Web of Science, without language restrictions between January 1, 2000 and March 31, 2019. Two authors independently extracted data and crosschecked the extraction to avoid errors. The quality of the included studies was examined following Cochrane guidelines.

**Results:**

We included 23 articles in our systematic review. The range of average override alerts was 46.2%-96.2%. An average of 29.4%-100% of the overrides alerts were classified as appropriate, and the rate of appropriateness varied according to the alert type (drug-allergy interaction 63.4%-100%, drug-drug interaction 0%-95%, dose 43.9%-88.8%, geriatric 14.3%-57%, renal 27%-87.5%). The interrater reliability for the assessment of override alerts appropriateness was excellent (kappa=0.79-0.97). The most common reasons given for the override were “will monitor” and “patients have tolerated before.”

**Conclusions:**

The findings of our study show that alert override rates are high, and certain categories of overrides such as drug-drug interaction, renal, and geriatric were classified as inappropriate. Nevertheless, large proportions of drug duplication, drug-allergy, and formulary alerts were appropriate, suggesting that these groups of alerts can be primary targets to revise and update the system for reducing alert fatigue. Future efforts should also focus on optimizing alert types, providing clear information, and explaining the rationale of the alert so that essential alerts are not inappropriately overridden.

## Introduction

### Rationale

A computerized provider order entry (CPOE) system is often integrated with a clinical decision support system (CDSS) to reduce patient harm and error rates [1]. A CDSS has immense potential for fostering patient safety and quality of care by reducing the adverse drug effects (ADEs) rate [1-3]. However, the current CDSS generates too many alerts, which are often overridden (approximately 90% to 95%), sometimes inappropriately. Concern related to inappropriate overrides reached a peak [4,5] with recognition of the potential to increase the risk of harm to patients. Multiple studies have reported that a high frequency of clinically irrelevant alerts (repetitive alerts with minimal clinical value), mediocre functionality (minimal integration among various departments and lack of alerts prioritization), and erroneous assessment by physicians are the main reasons for inappropriate overrides [4,6,7]. However, the growing number of inappropriate overrides often silently puts patients at risk of fatal ADEs [8,9].

To date, significant efforts have been taken to make sound clinical decisions and provide high-quality services. Indeed, lower specificity (high false-positives) and ambiguous alert contents (no clear information provided on why alerts were triggered in the systems) are still associated with excessive overrides and alert fatigue [4,10]. A CDSS with higher sensitivity and lower specificity could also contribute to the substantial number of inappropriate alerts [11,12]. Recent findings suggest that applying hard-stop alerts might be an efficient and helpful tool to reduce inappropriate overrides; however, such a tool must be judiciously implemented to achieve improved usability and receptivity of systems [13]. To increase the alert acceptance rate and reduce overrides, a system should be implemented in such a way that enables prioritizing alerts based on grade and potential harm, analyze the physician response, provide clear recommendations, and explain why the alert is triggered [14].

### Goal of Investigation

Since the override rate has been increasing, it is necessary to ascertain the types of alerts that are most frequently triggered, calculate the rate at which they are overridden (ie, reject the alerts), and to determine the reasons for overrides and the appropriateness of the reasons. Gaining a better understanding of these issues can provide meaningful insight into how alerts can be delivered in a relevant way (ie, converting a hard alert to a soft alert or turning off clinically irrelevant alerts or those with low clinical value).

## Methods

### Overview

**Figure 1:**
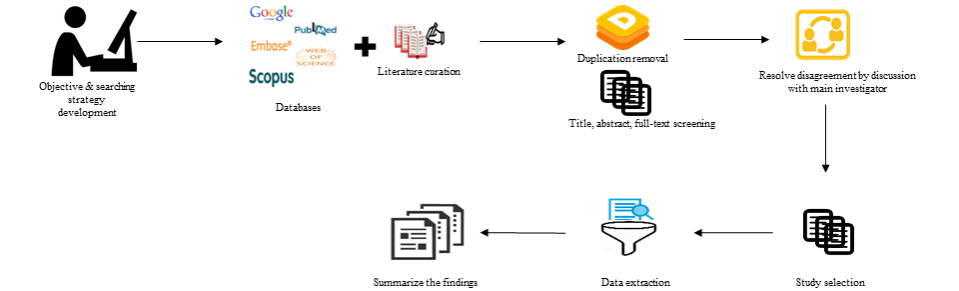
Overview of the study process.

We conducted a systematic review in accordance with the Meta-analysis of Observational Studies in Epidemiology guidelines [15] and the Preferred Reporting Items for Systematic Reviews and Meta-Analyses (PRISMA) standard [16]. The overview of the study process is given in Figure 1.

### Electronic Databases Search

We conducted a systematic search in electronic databases, including the PubMed, Embase, Scopus, Google Scholar, and Web of Science databases, between January 1, 2000 and April 30, 2019. The search was performed by two authors (MI and TP) using the keywords “alert fatigue,” “override alerts,” “computerized physician order entry,” “decision support system,” “medication-related CDS,” “CDSS,” and “CPOE.” There was no language and data restriction applied in the initial search. We also scanned the references of review articles and conference proceedings.

### Eligibility Criteria

The titles and abstracts of all retrieved studies were screened independently by two expert authors (MI and TP) to find the most relevant articles. They selected potentially eligible full-text articles. The full-text articles were considered as appropriate for inclusion in the systematic review by these same two experts after screening the full text and documenting the reasons for exclusion of inappropriate/ineligible articles. Any disagreement that arose in this screening process was resolved by the principal investigator of the study (YL). Articles were considered for inclusion if they met the following criteria: (1) published in English with desired outcomes reported, (2) evaluated override alerts along with reasons for those overrides, and (3) reported the override rate and the appropriateness of the override reasons.

We excluded studies if they were published in the form of a review, report, short communication, letter to editor, methodology, or editorial.

### Data Extraction

For studies that fulfilled the inclusion criteria, two authors (MI and TP) conducted data abstraction using a predefined, standardized protocol. Review Manager software (RevMan-5, Cochrane, UK) was also used to check the accuracy of the included studies. The following information was collected from the included studies: (1) methods, including setting, data analyzed, study design, study period, type of alerts, appropriateness criteria, inclusion and exclusion criteria; (2) results, including number of alerts, percent of override alerts, percentage of different types of alert overrides, percentage of overall override alerts, reasons for those overrides, characteristics of alert types, rate of appropriateness, rate of appropriateness for each override alert subtype, and rate of adverse effects; and (3) discussion, including the main findings, suggestions, intended recommendations, and limitations.

### Outcome Parameters

The following three primary outcomes were considered in our analysis: (1) characterize the types of alerts and their override rate; (2) the reasons for an override for different types of alerts assessed for inpatient and outpatient settings; and (3) the rate of the appropriateness of the override reasons.

## Results

### Literature Selection

**Figure 2:**
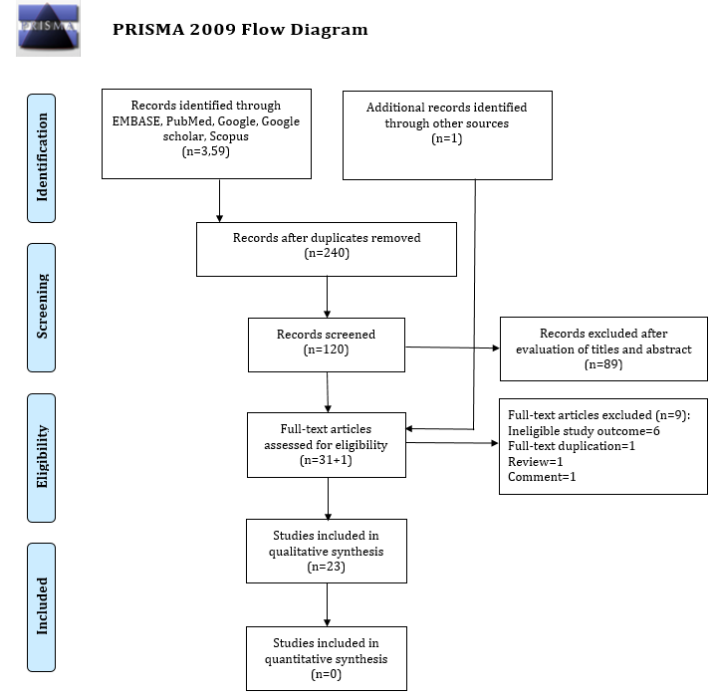
Preferred Reporting Items for Systematic Reviews and Meta-Analyses (PRISMA) flow diagram for study selection.

Our systematic search identified 360 titles and abstracts of potentially eligible studies for inclusion. Of these, 240 articles were excluded due to duplication and 88 of the remaining 120 articles were excluded based on predefined eligibility criteria during screening of titles and abstracts. The remaining 32 articles were processed for full-text review. Among these, a total of 23 relevant studies met all inclusion criteria [4,5,7,8,11,17-34]. Figure 2 shows all inclusion and exclusion criteria based on the PRISMA guidelines.

### Study Characteristics

The study characteristics of the included 23 articles are presented in Table 1. In this systematic review, six studies were based on a retrospective observational study design, five studies were cross-sectional, five studies were prospective observational studies, and seven studies only mentioned an observational study design. The settings included the intensive care unit (n=4), primary teaching hospital (n=5), academic medical center (n=5), tertiary care teaching hospital (n=3), university pediatric hospital (n=2), and others (n=4). There were 12 different types of alerts (drug-allergy, drug-drug interaction, drug-class, class-class, drug-dose, drug-duplication, drug-laboratory, drug-disease, drug-pregnancy, geriatric, age-based suggestion, renal, and formulary substitution) discussed in the included studies. The maximum override rate was 96.2%. ADEs were evaluated in 5 of the 23 studies [7,20,21,32,33].

**Table 1:** Characteristics of included studies.

Reference	Design	Setting	Period	Alert type	Override (%)	ADEs^a^ due to inappropriate override
Wong et al [7]	POS^b^	ICU^c^	September 2016-April 2017	Dose-range	93	Increased
Wong et al [17]	POS	In- and outpatients	January 2009-December 2011	DAI^d^	Inpatients, 46; outpatients, 68.8	N/A^e^
Cho et al [18]	ROS^f^	TAH^g^	September 2014-December 2014	DDI^h^	89.4	N/A
Nanji et al [19]	CSS^i^	TCTI^j^	2009-2012	DAI, DDI, DD^k^, ABR^l^, RR^m^, FS^n^	46.2	N/A
Wong et al [20]	POS	ICU	July 2016-April 2017	DAI, DDI, geriatric, renal	88.5	Increased
Rehr et al [8]	POS	ICU	June 2016- November 2016	Dose, DDI, DAI	66.0	N/A
Wong et al [21]	ROS	ICU	2009-2011	DAI, DDI, geriatric, renal	~87.1	Increased
Slight et al [22]	CSS	TCTI	January 2009- December 2011	DAI	81.0	N/A
Topaz et al [23]	RCSS^o^	AMC^p^	2004-2013	DAI	87.6	N/A
Her et al [24]	OS^q^	AMC	January 2012-December 2012	NFM^r^	~61.2	N/A
Topaz et al [25]	OS	AMC	2004-2013	DAI	89.7	N/A
Straichman et al [5]	OS	AMC	November 2013-December 2013	Dose, RDA^s^, DT^t^, DDI	96.2	N/A
Ahn et al [26]	ROS	ED^u^ and GW^v^	September 2009-June 2013	DDI	ED: 94 GW: 57	N/A
Nanji et al [4]	OS	OP^w^ and AHBP^x^	Jan 2009-December 2011	DAI, DDI, DD, DCI^y^, CCI^z^, ABS^aa^, RS^bb^, FS	52.6	N/A
Cho et al [27]	CSS	OP	January 2009-December 2011	Renal dose	78.2	N/A
Bryant et al [28]	ROS	PTH^cc^	June 10-13, 2013	DDI	~95.1	N/A
Jani et al [34]	ROS	AMC	October 2005-October 2006	DAI, DDI, DT	89.0	N/A
Slight et al [11]	CSOS^dd^	PTH	January 2009-December 2011	DDI	53.4	N/A
Mille et al [29]	POS	UPH^ee^	November 2006-December 2006	DDI	68.7	N/A
Van der Sijs et al [30]	ROS	UPH	2001-2005	DDI	72.0	N/A
Shah et al [31]	OS	PTH	August 2004- January 2005	DD, DDI, DL^ff^, DID^gg^, DP^hh^	71	N/A
Hsieh et al [32]	OS	PTH	August 2002-October 2002	DAI	80.0	Increased
Weingart et al [33]	OS	PTH	October 2000- December 2000	DDI, DAI	~91.2	Increased

^a^ADE: adverse drug effect.

^b^POS: prospective observational study.

^c^ICU: intensive care unit.

^d^DAI: drug-allergy interaction.

^e^N/A: not applicable.

^f^ROS: retrospective observational study.

^g^TAH: tertiary academic hospital.

^h^DDI: drug-drug interaction.

^i^CSS: cross-sectional study.

^j^TCTI: tertiary-care teaching hospital.

^k^DD: duplicate drug.

^l^ABR: age-based recommendation.

^m^RR: renal recommendation.

^n^FS: formulary substitution.

^o^RCSS: retrospective cross-sectional study.

^p^AMC: academic medical center.

^q^OS: observational study.

^r^NFM: nonformulary medication.

^s^RDA: renal dose adjustment.

^t^DT: duplicate therapy.

^u^ED: emergency department.

^v^GW: general ward.

^w^OP: outpatients.

^x^AHBP: ambulatory hospital-based practice.

^y^DCI: drug-class interaction.

^z^CCI: class-class interaction.

^aa^ABS: age-based suggestion.

^bb^RS: renal suggestion.

^cc^PTH: primary teaching hospital.

^dd^CSOS: cross-sectional observational study.

^ee^UPH: university pediatric hospital.

^ff^DL: drug lab.

^gg^DID: drug-disease.

^hh^DP: drug pregnancy.

### Appropriateness Criteria

**Figure 3:**
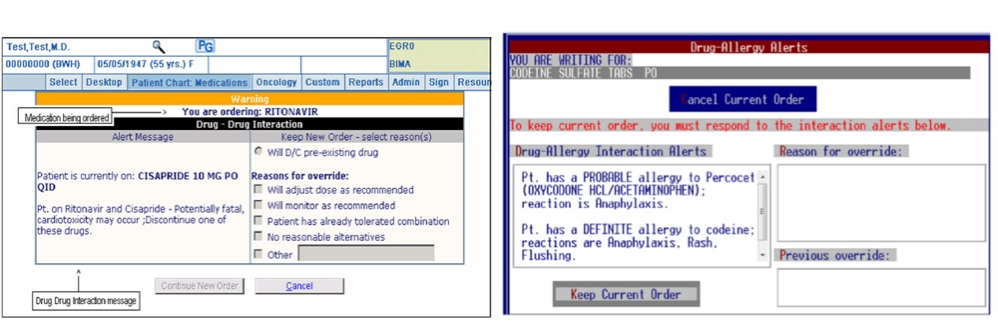
Example of a drug-drug interaction alert (left) and drug-allergy interaction alerts (right).

All of the included studies developed criteria for evaluating the appropriateness of overrides for each alert type for both inpatient and outpatient settings. To validate the appropriateness framework, they used a chart along with previously published articles and clinical experience of the multidisciplinary group (physicians, pharmacists, and nurses). All studies used specific criteria for different types of alerts, which were modified until reaching a final agreement. They considered override alerts as appropriate if the reasons reported by the physicians were acceptable according to their study’s framework and also verified based on review of relevant guidelines. For example, if a clinician prescribed a drug and a dose alert was displayed, the appropriate override reasons mentioned were “will monitor as recommended,” “will adjust the dose,” and “patient has already tolerated” based on previous data, indicating that monitoring is beneficial to patients (Figure 3). Subsequently, the multidisciplinary group carefully checked and verified all of the override reasons in their chart review. They extensively verified by reviewing guidelines, such as checking for dose/renal function/drug monitoring criteria, previously prescribed tolerable medication combinations, and accepted/refused medication. The included studies mentioned that pharmacists, nurses, training health care personal, and clinicians checked and verified the appropriateness of override reasons. Any disagreements among them were resolved with discussion.

### Override Rate and Appropriateness of Overrides

All 23 studies described the alert override rate and the appropriateness of overrides according to alert type (drug-allergy, drug-drug, dose, drug-class, class-class, drug duplication, drug-laboratory, drug-disease, drug-pregnancy, geriatric, renal-dose, age-based suggestion, renal, formulary substitution). The average override alerts ranged from 46.2% to 96.2% (Table 1). However, the range of override rates varied according to alert type (drug-allergy 46%-95%, drug-drug interaction 56.3%-95.6%, dose 82%-96.8%, geriatric 2.1%-87.1%, and renal 74.4%-97.1%). Moreover, the overall appropriateness rate ranged from 29.4% to 100%, which also varied according to alert types (drug-allergy 63.4%-100%, drug-drug interaction 0%-95%, dose 43.9%-88.8%, geriatric 14.3%-57%, renal 27%-87.5%). However, interrater reliability for the assessment of override alerts appropriateness was excellent (kappa=0.79-0.97). Table 2 summarizes the rates of override alerts and the appropriateness of override alerts by type.

**Table 2:** Rate of override alerts and appropriateness of override alerts by type.

Reference	Type of alerts	Overridden (%)	Appropriateness (%)	Evaluation criteria	Evaluation rater	Interrater agreement, kappa (95% CI)
Wong et al [7]	Dose	93.0	88.8	Based on previously published data including guidelines	Clinical pharmacist and research assistant	0.87 (0.85-0.90)
Wong et al [17]	DAI^a^	Inpatient: 46.0; outpatient: 68.8	Inpatient: 83.9; outpatient: 100	NR^b^	NR	NR
Cho et al [18]	DDI^c^	71.7	~75.3	Based on previously published data including guidelines	Physicians	0.92
Nanji et al [19]	DAI, DDI, and DD^d^	DAI: 81.9, DDI: 68.2, DD: 51.9	DAI: 96.5, DDI: 62.0, DD: 98.0	Based on previously published data including guidelines	Physician and pharmacist	0.96 (0.95-0.97)
Wong et al [20]	DAI, DDI, dose, geriatric, renal	DAI: 83.6, DDI: 91.9, dose: 96.8, geriatric: 2.30, renal: 97.1	DAI: 83.4, DDI: 82.0, dose: 43.9, geriatric: 14.3, renal: 87.5	Based on previously published data including guidelines	Clinical pharmacist	0.89 (0.85-0.93)
Rehr et al [8]	DAI, DDI, dose	DAI: ~80, DDI: ~87, dose: ~82	DAI: 83.0, DDI: 0.00, dose: 85.0	NR	NR	NR
Wong et al [21]	DAI, DDI, geriatric, renal	DAI: 46.3, DDI: 56.3, geriatric: 87.1, renal: 74.4	DAI: 94.0, DDI: 84.0, geriatric: 57.0, renal: 27.0	Based on previously published data including guidelines	Clinical pharmacist	0.79 (0.73-0.86)
Slight et al [22]	DAI	Inpatient: 83.0; outpatient: 81.0	Inpatient: 96.5; Outpatient: 94.0	Based on previously published data including guidelines	Pharmacist	0.86
Topaz et al [23]	DAI	~87.6	NR	NR	NR	NR
Her et al [24]	FA^e^	~61.2	82.8	Based on previously published data including guidelines	Pharmacist	0.97 (0.92-1.00)
Topaz et al [25]	DAI	89.7	NR	NR	NR	NR
Straichman et al [5]	Dose, RDA^f^, DT^g^, DDI, MDDI^h^	Dose, 92.1; RDA: 92.3, DT: 96.0, DDI: 95.6, MDDI: 96	Overall: 84.5	Based on previously published data including guidelines	Pharmacist	NR
Ahn et al [26]	DDI (ED^i^), DDI (GW^j^)	DDI (ED): 94.0, DDI (GW): 57.0	DDI (ED): 59.6, DDI (GW): 40.4	NR	NR	NR
Nanji et al [4]	DAI, DDI, DD^f^, DCI^k^, CCI^l^, ABS^m^, renal, FA	DAI: 77.4, DDI: 60.2, DD: 28.6, DCI: 24.4, CCI: 69.7, ABS: 79.0, renal: 78.0, FA: 85.0	DAI: 92.0, DDI: 12.0, DD: 82.0, DCI: 88.0, CCI: 69.0, ABS: 39.0, renal: 12.0, FA: 57.0	Based on previously published data including guidelines	Physician, pharmacist, and nurse	0.89
Cho et al [27]	Renal	78.2	29.4	Based on previously published data including guidelines	Physician	0.93
Jani et al [34]	DAI, DDI, DT, ED	DAI: 63.4, DDI: 73.0, DT: 95.0, ED: 90.6	NR	NR	NR	NR
Bryant et al [28]	DDI and DAI	DDI: 95.0 and DAI: 91.0	NR	NR	NR	NR
Slight et al [11]	DDI	60.0	68.2	Based on previously published data including guidelines	Pharmacist	0.84
Mille et al [29]	DDI	68.7	NR	NR	NR	NR
Van der Sijs et al [30]	DDI	72.0	NR	NR	NR	NR
Shah et al [31]	DCI, DDI, DLI^n^, DRDI^o^, and DPI^p^	DCI: 23.0, DDI: 58.0, DLI: 60.0, DRDI: 47.0, DPI: 90.0	NR	NR	NR	NR
Hsieh et al [32]	DAI	80.0	NR	NR	NR	NR
Weingart et al [33]	DAI and DDI	DAI: 91.2; DDI: 94.6	63.5	Based on previously published data including guidelines	Board-certified internee	0.86

^a^DAI: drug-allergy interaction.

^b^NR: not reported.

^c^DDI: drug-drug interaction.

^d^DD: drug duplicate.

^e^FA: formulary alert.

^f^RDA: renal dose adjustment.

^g^DT: duplicate therapy.

^h^MDDI: major drug-drug interaction.

^i^ED: emergency department.

^j^GW: general ward.

^k^DCI: drug-class interaction.

^l^CCI: class-class interaction.

^m^ABS: age-based suggestion.

^n^DLI: drug-lab interaction.

^o^DRDI: drug-disease interaction.

^p^DPI: drug-pregnancy interaction.

### Reasons for Overrides

All 23 included studies evaluated the reasons for overriding the alerts in the CDSS. The most common reasons for overriding drug-allergy alerts were “will monitor,” “patients tolerated before,” “patient took previously without an allergic reaction,” “low risk across sensitivity,” “no reasonable alternatives,” and other (with or without a free-text reason provided). The most common reasons for overriding drug-drug interaction alerts were “will monitor as recommended,” “will adjust the dose as recommended,” “patients have already tolerated this combination,” “clinically irrelevant,” and “benefit assessed to be greater than the risk.” Moreover, the common reasons for overriding dose alerts were “will adjust the dose as recommended,” “benefit outweighs risk,” and “patients tolerated before” (Table 3).

**Table 3:** Override reason by alert type.

Alert type	Override reason
Drug-allergy	Will monitor [4,8,19,20,25,32,34]. Patient tolerated before [8,20,21,25,32-34]. Patient took previously without allergic reaction [4,5,17,19-22,32,34]. Provider approved [28]. Low risk across sensitivity [4,19,21,22]. No reasonable alternatives [4,22,25,33]. Limited course of treatment [33]. Physician aware [17,19,21,22,32,34]. Alerted interaction not clinically significant [28]. Allergy information inaccurate in patient’s records [33]. Patient does not have this allergy, will D/C the pre-existing allergy [17,25,32]. Desensitization [17]. Administer per desensitization protocol [17]. Other (allows user to enter free text) [8,22,25,30,33]. Other (with no free-text reason provided) [4,22,33]. Unknown [21,23,25,30].
Drug-drug interaction	Will monitor as recommended [4,5,8,11,19-21,31,34]. Will adjust dose as recommended [4,8,11,19,21,31]. Patient has already tolerated this combination [5,8,11,19,21,31,33,34]. No reasonable alternative [4,11,31,33]. Clinically irrelevant alert [5,26,28,30,33]. Medication list out of date [33]. Limited course of treatment [4,33]. Benefit assessed to be greater than the risk [5,26,29,33]. The drug combination will be given only for a short period and is therefore safe [5]. The computerized system did not interpret my prescription correctly [5]. The drug-drug interaction is unlikely to occur because of the route of administration [5]. Combinations of the coded reasons listed above [11,30]. Other (allows user to enter free text) [4,11,26,30,33]. Other (with no free-text reason provided) [4,11,19,33].
Drug-class	Will monitor as recommended [4]. Will adjust the dose as recommended [4]. Patient has already tolerated this combination [4]. No reasonable alternatives [4]. Others [4].
Class-class	Will monitor as recommended [4]. Will adjust the dose as recommended [4]. Patient has already tolerated this combination [4]. No reasonable alternatives [4]. Others [4].
Drug-dose	Will adjust dose as recommended [5,7,8,20]. Benefit outweighs risk [5,7]. Patients tolerated before [5,7]. Inaccurate warning [7,8]. The drug combination or the drug at the given dose before without any adverse effects [5]. The drug dose alert is based on patient weight which is unavailable in the electronic patient record [5].
Drug-duplication	Combination therapy indicated [19]. One-time dose [19]. Not duplicate therapy [19]. Patient requires different strengths of the same drug [4,5]. Transitioning from one drug to the other [4,31]. Patient on long-term therapy with a combination [4,31]. Advice from a consultant [4]. New evidence supports duplicate therapy of this type [4]. Others [4].
Drug-lab	Will monitor/manage as recommended [31]. More recent lab results available that warrant use [31].
Drug-disease	Patient has tolerated this drug in the past [31]. New evidence supports the therapy of this type [31].
Drug-pregnancy	Patient is not pregnant [31]. Patient is not of child-bearing potential [31]. Advice from a consultant [31]. No reasonable alternative [31]. Patient has tolerated in the past [31]. Medication is for short-term/as-needed use only [31].
Geriatric	Patient tolerated before [21]. Will monitor later [20].
Age-based suggestion	Patient has tolerated this drug in the past [4]. Advice from a consultant [4]. New evidence supports the therapy of this type [4]. Others [4].
Renal suggestion	Will monitor as recommended [5,20,21]. Patient has tolerated this drug in the past [4,5,21,27]. New evidence supports the therapy of this type [4,27]. Advice from a consultant [4,27]. The computerized system did not interpret my prescription correctly [5]. Others [4,27].
Formulary substitution	Intolerance/failure of suggested substitution [4,24]. Patient preference [4]. Patients currently taking prescribed medication [4]. Insurance does not allow the above suggestion [4]. Written originally by another physician [4]. Pharmacological [24]. Specialist recommendation [24]. Disease or condition [24]. Blank [24]. Others [4].

### ADEs

Five studies compared ADEs based on an appropriate and inappropriate override. Wong et al [17] demonstrated a significantly increased rate of ADEs in inappropriate override dose alerts compared with appropriate override dose alerts. The rate of ADEs per 100 override dose alerts was 1.3 and 5.0 for appropriate and inappropriate override dose alerts, respectively. Wong et al [20] also evaluated the potential and definite ADEs in 5 different types of alerts reported, demonstrating that the average potential and definite ADEs were higher in alerts that were considered to be inappropriately overridden than appropriate override alerts (16.5 vs 2.74 per 100 overridden alerts, *P*<.001). However, the rate of ADEs was always higher for inappropriate override alerts (drug-allergy interaction: 11.5 vs 0.6; drug-drug interaction: 11.4 vs 2.0; dose: 17.6 vs 11.1; geriatric: 11.1 vs 0; and renal: 30.8 vs 0). The logistic regression model showed that inappropriate override alerts were significantly associated with an increased risk of ADEs (odds ratio 6.14, 95% CI 4.63-7.71, *P*<.001) and an increased intensive care unit length of stay (2.25 days, 95% CI 0.52-3.98, *P*=.01). Moreover, 3 studies also reported that inappropriate override was associated with an increased risk of ADEs [21,32,33].

## Discussion

### Main Findings

This is the first systematic review that evaluated the current scenario of a CDSS by measuring the rate with which alerts are overridden, described the reasons for override alerts at the time of prescribing, and the appropriateness of overrides. A significant proportion of alerts in the CDSS were overridden (96.2%), and the override rate varied dramatically according to alert types. The rate of appropriate overrides was high (nearly 100%) and they also varied significantly according to alert types. For example, renal, geriatric, and drug-drug interaction alert overrides had low appropriateness rates, whereas drug-allergy, drug-duplication, drug-formulary, and drug class alert overrides had higher appropriateness rates. Inappropriate overrides were associated with an increased risk of ADEs when compared with appropriately overridden alerts. However, the reasons provided for overriding alerts varied extensively depending on alert types. Refinement of these alert types has immense potential to improve the acceptance rate and patient safety. Furthermore, the clinical team should evaluate the appropriateness of overrides based on the given clinical context to optimize alert types and frequencies and ultimately improve their clinical relevancy while reducing alert fatigue.

### Clinical Implications

A CPOE integrated with a CDSS is designed to improve patient safety and reduce preventable errors by generating pop-up alerts at the point of order entry. A frequent complaint about CPOEs is firing up too many alerts, which are frequently not clinically relevant or have very low clinical value [35]. An excessive number of alerts in the CPOE desensitizes physicians (hampers the mental state, consumes too much time), leading them to override both appropriate and clinically irrelevant alerts [36]. A system with low sensitivity (ie, more false-negatives) and low specificity (ie, more false-positives), ambiguous information content, and an overwhelming number of alerts (both relevant and irrelevant alerts) induce alert fatigue [35]. Inappropriate override always leads to potential ADEs and increased morbidity [37]. A study evaluating drug-drug interaction alert overrides and how override alerts lead to preventable ADEs reported 22 serious ADEs over the 3-month study period [32].

In our study, a high number of alerts were overridden, especially for dose, drug-drug interaction, and drug-allergy interaction alerts. The findings of our study also suggest that a higher number of these alerts can lead to alert fatigue. There are two ways to combat alert fatigue. First, the system should set a higher threshold for triggering alerts. Second, the most frequent alerts should be categorized and the system updated regularly (overrides tend to increase over time). We also evaluated the appropriateness of alert overrides, demonstrating that the rate of the appropriateness of overrides varied according to the different types of alerts. Evaluating the appropriateness of override alerts is difficult but the range of interrater reliability for assessment was high. Among the dose recommendation alerts that were overridden, only 43.9%-85% were found to be appropriately overridden. The range of appropriately overridden renal and drug-drug interaction alerts was 12%-87.5% and 0%-84%, respectively. Among the overridden drug-allergy interaction recommendation alerts, approximately 83.5%-100% were appropriately overridden. Moreover, the vast majority of drug-duplicate (82%-99%), drug-class (88%), and formulary (82.8%) override alerts were appropriate, indicating that these groups can be the primary targets for rectification to stop alert fatigue by reducing or converting (hard-stop alert to soft/passive alerts) the number of alerts. However, the higher rates of inappropriate overrides of the renal, drug-drug interaction, and geriatric alert types indicate the need for further intervention.

Our findings also provide a variety of reasons for overriding alerts. The majority of physicians provided the reasons for overrides as “will monitor as recommended,” “patients have tolerated it before,” “will adjust the dose,” and “maximum time,” leaving the free-text box blank. In some cases, physicians do not write any reason for the overrides; however, it is important to clearly outline the override reasons to best invest in the patient’s condition and care. Indeed, these findings raise concern about patient safety and quality of care. For example, failure to monitor several drug levels such as digoxin after initiation of verapamil (drug-drug interaction) can cause serious harm for the patient [38]. Other common reasons physicians gave for the override were “no reasonable alternative,” “physician aware,” “patients have already tolerated this combination,” and other (without free-text reason provided). However, there was no confirmation that physicians were actually aware of the potential harm and had monitored the patient’s condition before overriding. Moreover, in the case of drug-allergy, approximately two-thirds of alerts that showed a reaction of “anaphylaxis” were overridden by physicians and with the reasons provided including *“*patients have taken previously without an allergic reaction” and “low-risk cross-sensitivity.” However, a patient with a true allergy can experience severe anaphylaxis. For example, a reaction between vancomycin and red man syndrome was found to be inappropriately overridden with the reason given that the “patient has taken previously without allergic reaction/patient has tolerated previously,” but severe ADE was observed (development of a patchy macular rash) [21]. Therefore, it is essential to know the patient’s history of anaphylaxis to reduce serious recurrence (approximately 35% of patients experience recurrence) [39]. Topaz et al [23] reported that only about one-tenth of the alerts showed potential life-threatening effects that were a definite match between the allergy and prescribed drugs, although others were due to either the “cross-sensitivity or allergy group.” Several studies confirmed that the hospitalization of patients with anaphylaxis has been increasing in both the United States [40] and the United Kingdom [41]. It is therefore important to evaluate these types of override alerts and the reasons given by the physician for the override. Moreover, future studies are needed to develop an effective knowledge management system that can provide more accurate and relevant drug-allergy interaction alerts for improving patient safety.

### Recommendations to Improve the CDSS

The findings of our study provide a clear picture of the overall situation of current CDSSs by summarizing the existing literature. These findings can help policymakers and researchers to improve existing CDSSs by conducting an in-depth analysis of existing CDSS features. Having provided a collection of evidence-based information and removing unimportant alerts, a novel system also requires rigorous evaluation to determine the optimum rate of sensitivity and specificity for reducing patient harm. No system can achieve 100% sensitivity and specificity in a real-world setting. However, a logical and effective symmetry between sensitivity and specificity can make the system more flexible and safer. The sensitivity and specificity should be increased without sacrificing the other through the combination of patient factors and using futuristic algorithms. Osheroff et al [42] demonstrated that “five rights” (right information, to the right person, in the right CDS intervention format, through the right channel, and at the right time in the workflow) should be taken into consideration when alerts will be popped up in the system. Several recommendations are provided below to design a sophisticated CDSS by reducing alert fatigue.

First, increase the positive predictive value for dose recommendation alerts by incorporating patient-specific factors (eg age, other medication orders, renal impairment history) [7].

Second, optimize alert types and frequencies to increase their clinical relevance so that important alerts are not inappropriately overridden [43].

Third, override alerts can be revised if they are not clinically important, and the system will be updated for reducing alert fatigue.

Fourth, turn off alerts that are not clinically important/inaccurate or of only minor importance [8,44].

Fifth, it is essential to categorize the most frequent interruptive alerts; for serious alerts such as drug-drug interactions and renal, the dose should be displayed as interruptive, whereas minor/low-risk alerts can be presented in a noninterruptive manner [13].

Sixth, all types of alerts should contain clear and concise information [45,46] and provide exact information on why the alert is important for the situation [47].

Seventh, identify a list of medications that patients previously showed no allergic reaction to or tolerated in the past so that physicians are not inundated with highly irrelevant alerts. Alerts to previously tolerated medications might be presented in a noninterruptive fashion [48-50].

Eighth, systems should pay more attention to the storage of override reasons data (eg, dose-range, allergy) [50,51], and encourage providers to provide accurate override reasons [52,53].

Ninth, identify the malfunctions and pattern of malfunctions in the CDSS [54].

Tenth, it is essential to remove the repetitive and duplicate nature of alerts in the CDSS [21,55].

Eleventh, it is important to understand the system behavior and patterns of physicians in accepting and rejecting the alerts [18].

Twelfth, the system can trigger an alert based on the specialty of physicians (eg, do not provide too many renal alerts for kidney specialists and those with many years of experience in this field) [30,56].

Thirteenth, a drug-drug interaction alert can be presented in an “alert tiering” based on the level of severity. For example, level-1, level-2, and level-3 will be considered as life-threatening, less serious, and least serious, respectively. For level-1 and level-2, hard-stop alerts will be applied, whereas a passive alert (no need for physicians action) can be applied [57].

Fourteenth, it is important to use hard-stop alerts for drug-drug interactions, renal, and geriatric alerts that might harm patients and to use soft-stop alerts for a formulary, drug-allergy, and drug-class alerts that have a lower risk for patient harm [57].

Fifteenth, review alerts periodically and improve according to clinical importance [58-60].

Sixteenth, always encourage physicians to provide override reasons. Learn from the override reasons and place maximum effort to improve the system [61].

Seventeenth, when designing the system, form a multidisciplinary committee consisting of physicians, pharmacists, information technology specialists, and quality administrators [62-64].

Finally, do not establish a silo alert system (always integrate multidepartment data) [62].

### Strengths and Limitations

There are several strengths of our study that should be mentioned. First, this is the very first systematic review that summarizes the overall override rate, reasons for overriding the alerts, and the appropriateness of the reasons. Second, we have also provided an override rate and the proportion of appropriateness according to various types of alerts. These data can help policymakers in determining the area that they should place more focus to reduce alert fatigue. Finally, we have provided recommendations to optimize alert types and to improve the clinical relevance of alerts while suppressing alert fatigue for the CDSS that is often injudiciously overridden.

Our study also has several limitations. First, we could not determine the bias of the included studies because of the heterogeneous nature of the studies. Second, we could not provide the percentage of ADEs when alerts were inappropriately overridden owing to data scarcity. Finally, some studies used a random sampling method of alert overrides reviewed for appropriateness that was very trivial compared with the entire alerts fired up in the CDSS, and the accuracy of such reviews was completely reliant on information contained in the patients’ charts. However, this may vary from study to study.

### Conclusion

The findings of our study show that a high proportion of alerts are overridden and the rate of appropriateness varies widely according to alert type. Although the CDSS is an extremely effective tool for reducing patient harm and improving quality of care, it could also diminish patient safety if information technology vendors and health care professionals do not appropriately design the clinical interface. Future research should be focused on how to obtain meaningful information for analyzing these override reasons and how to integrate patient-specific factors to reduce alert fatigue, resulting in a more efficient, safe, and effective system.

## Abbreviations

ADE: adverse drug effects
CDSS: clinical decision support system
CPOE: computerized provider order entry
PRISMA: Preferred Reporting Items for Systematic Reviews and Meta-Analyses
